# Complexity, Predictability and Time Homogeneity of Syntax in the Songs of Cassin’s Vireo (*Vireo cassinii*)

**DOI:** 10.1371/journal.pone.0150822

**Published:** 2016-04-06

**Authors:** Richard W. Hedley

**Affiliations:** Department of Ecology and Evolutionary Biology, University of California Los Angeles, Los Angeles, California, United States of America; Texas Christian University, UNITED STATES

## Abstract

Many species of animals deliver vocalizations in sequences presumed to be governed by internal rules, though the nature and complexity of these syntactical rules have been investigated in relatively few species. Here I present an investigation into the song syntax of fourteen male Cassin’s Vireos (*Vireo cassinii*), a species whose song sequences are highly temporally structured. I compare their song sequences to three candidate models of varying levels of complexity–zero-order, first-order and second-order Markov models–and employ novel methods to interpolate between these three models. A variety of analyses, including sequence simulations, Fisher’s exact tests, and model likelihood analyses, showed that the songs of this species are too complex to be described by a zero-order or first-order Markov model. The model that best fit the data was intermediate in complexity between a first- and second-order model, though I also present evidence that some transition probabilities are conditioned on up to three preceding phrases. In addition, sequences were shown to be predictable with more than 54% accuracy overall, and predictability was positively correlated with the rate of song delivery. An assessment of the time homogeneity of syntax showed that transition probabilities between phrase types are largely stable over time, but that there was some evidence for modest changes in syntax within and between breeding seasons, a finding that I interpret to represent changes in breeding stage and social context rather than irreversible, secular shifts in syntax over time. These findings constitute a valuable addition to our understanding of bird song syntax in free-living birds, and will contribute to future attempts to understand the evolutionary importance of bird song syntax in avian communication.

## Introduction

Bird song ranges from very simple to highly variable and complex [1]. The preferred measure of song complexity has traditionally been repertoire size [2,3], but this is just one way to consider the topic. Many species, for example, appear to deliver their songs according to highly structured sequencing rules, or syntax, effectively increasing their apparent complexity while maintaining modest repertoire sizes [4–6]. Though various studies have presented evidence for this type of non-random syntactic structure in the songs of numerous bird species [5–14], only a few have attempted to rigorously classify the statistical complexity of songbird syntax [15–17], and these have been restricted to captive birds. The paucity of empirical results regarding the nature of bird song syntax restricts our understanding of the functional importance of syntax in communication and the extent of variation possible in this trait. In particular, various authors have suggested that the syntactic rules employed by non-human animals appear to be constrained in their sophistication [18–20], a hypothesis that can only be evaluated with additional analyses from a broader array of species. This paper assesses the syntactic complexity of the songs of a population of free-living Cassin’s Vireos, contributing to a growing understanding of bird song syntax and providing a foundation for future studies of the potential role of syntax in the conveyance of information in this species.

Studies of bird song syntax have typically relied upon Markov models to evaluate their complexity, with the complexity of syntax relating to the complexity of the model that best fits the observed sequences [6,8–12,15,16]. This approach shares similarities with the concept of algorithmic complexity in computer science, where the complexity of a sequence of symbols–in this case, a sequence of bird songs–is analogous to the length of the shortest computer program that can describe it [21]. Algorithmic complexity is probably too theoretical to be of use for applications in biology, however, because even simple biological systems with uncomplicated rules can produce phenomena whose properties are immensely complex [22]. For this paper, I shall adopt a definition from statistical learning theory, which relates model complexity to the number of independent parameters in the model [23]. In the case of Markov models, I consider the complexity of the model to be equivalent to the number of states in the model [24]. Simple syntax, therefore, produces sequences that can be described by a zero-order or first-order Markov model [7], while sequences generated by more complex syntax can be described by either higher-order Markov models [6,8,12] or variants thereof, such as Prediction Suffix Trees [17], hidden Markov models [15], or partially-observable Markov models [16]. The latter models are characterized by larger numbers of model states allowing them to model non-adjacent dependencies in sequences, where the identity of upcoming vocalizations depends on more than one preceding vocalization.

Cassin’s Vireos sing elaborate sequences of phrases that, when recorded over long periods, show extensive evidence of repeated syntactic patterns [25]. The species also displays high levels of interannual site fidelity, non-overlapping territories, and prolific vocal output, making them an ideal species in which to study the sequential complexity of songs. Here I present analyses fitting Markov models to the songs of a wild population of Cassin’s Vireos from California, to assess the complexity of the song syntax and to investigate the existence of non-adjacent dependencies in the songs of the species.

## Results

### Song characteristics

**Fig 1:**
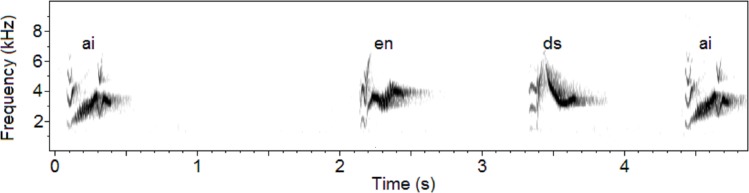
Spectrogram of four phrases from the songs of the ‘Gully’ individual. The sequence depicted shows the phrase types *ai*, *en*, *ds*, and *ai*. The clear resemblance of the first and last phrases illustrates the high levels of within-type stereotypy in the songs of the species.

I recorded the songs of fourteen male Cassin’s Vireos over two breeding seasons in 2013 and 2014 (see Materials and Methods). Males in this species sing short phrases at varying rates throughout the day. Each phrase can be classified as a phrase type based on its acoustic features (Fig 1; S1 File), and each individual possesses a repertoire containing an average of 51 phrase types; repertoire sizes and summary statistics of the recording corpora for each individual can be found in Table 1. Singing bouts are not well defined in this species: although their modal song rate is approximately one phrase every two seconds, they often sing at lower, yet steady, rates. Accordingly, each recording was analyzed as a single sequence, regardless of the durations of silence contained therein. A more detailed description of this species’ singing behavior can be found in Hedley [25].

**Table 1:** Summary of each individual’s recording corpus, along with the results from the Fisher’s tests for second- and third-order dependencies.

Individual	No. recordings	Phrases recorded	Repertoire size	Fisher’s Exact Tests			
				Order of dependency	No. comparisons (n)	Significant at p<0.05	Significant at p<0.05/n
AGBk	34	10336	55	2	51	25	17
				3	222	22	5
AGO	22	4492	52	2	46	19	11
				3	151	12	1
AOBu	20	6983	59	2	52	16	10
				3	243	14	3
AYO	9	1335	46	2	41	8	2
				3	81	0	0
BuRA	19	4919	51	2	49	18	8
				3	251	8	0
Gate	9	1570	45	2	37	9	1
				3	97	2	0
GRA	11	3918	60	2	55	24	9
				3	166	15	1
Meadow	7	1505	44	2	43	8	3
				3	95	1	0
ORA	11	2262	46	2	34	16	9
				3	89	7	2
RYA	14	3037	47	2	44	15	6
				3	144	4	0
Gully	4	2322	47	2	46	23	6
				3	156	3	0
WABk	30	8588	56	2	54	31	14
				3	235	11	3
YAW	15	3166	52	2	46	11	4
				3	145	7	1
YBuA	16	2944	52	2	48	18	6
				3	165	6	1

### Simulations

Sequence simulations are a common technique used to assess the syntactic rules of bird song, since they are a simple method for generating sequences from models with known complexity which can then be compared with the observed sequences derived from a bird’s internal song system with unknown complexity [9,26]. The methods used here were broadly similar to those of Jin and Kozhevnikov [16], in that I divided each bird’s song corpus into a training set for parametrization and a testing set held out for comparison. I used a zero-order, first-order and second-order Markov model to simulate song sequences, and the resulting sequences and the training set were compared to the testing set, by comparing N-gram distributions and recurrence interval distributions with those of the testing set using L_1_-distances (see Materials and Methods). If the differences, reflected in the L_1_-distances, between the simulated data and the testing set were comparable in magnitude to the differences between the training set and the testing set, this would indicate that the model used to generate the simulations was similar to the syntax employed by the bird itself, or at least that their output shared similar properties.

**Fig 2:**
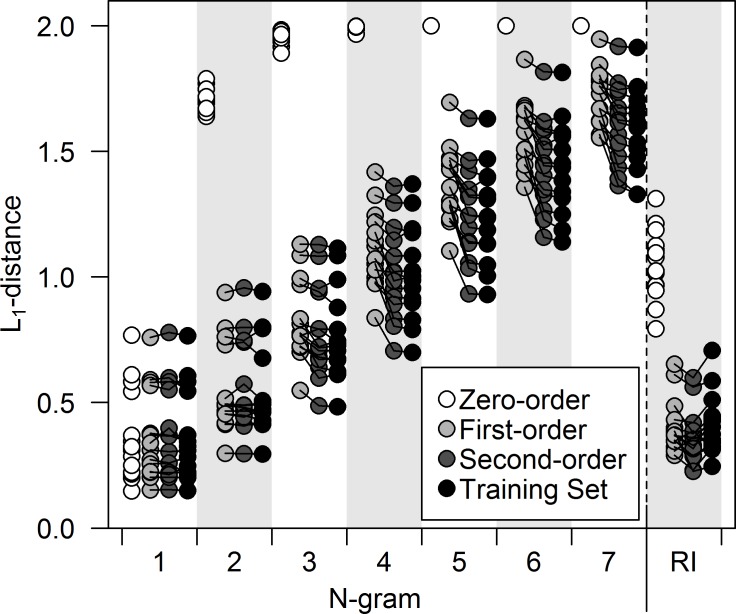
L_1_-distances for the three candidate models compared with those of the training set. Lines connect results from a given individual for the first-order, second-order, and training results, but were not drawn from the zero-order model for clarity and because this model so clearly diverged from the rest. Values on the left represent the seven N-gram distributions, while the values on the right represent the recurrence interval (RI) distributions.

The simulations suggested that the second-order Markov model closely approximated the singing style for all individuals (Fig 2). At N = 1, the N-gram distribution for all models did not differ from the L_1_-distances for the training set (one-tailed paired t-tests vs training set: zero-order, p = 0.13; first-order, p = 0.27; second-order, p = 0.25). At N≥2, the L_1_-distances for the zero-order model diverged from the expected distribution (one-tailed paired t-tests vs training set, p<0.001 at N = 2 thru N = 7), illustrating that the sequences generated by the zero-order model differed drastically from the sequences upon which they were parametrized. As the N-gram increased, the L_1_-distances for the first-order model diverged from the values for the training set (one-tailed paired t-tests vs training set, p = 0.28 at N = 2, p<0.001 at N = 3 to N = 7), while the values for the second-order model remained similar to those of the training set until N = 6, only showing a significant difference at N = 7 (Fig 2; one-tailed paired t-tests vs training set, p = 0.27 at N = 2, p = 0.23 at N = 3, p = 0.54 at N = 4, p = 0.20 at N = 5, p = 0.081 at N = 6, p = 0.003 at N = 7). The first- and second-order models both produced a similar recurrence interval distribution to that described by Hedley [25], biased towards low recurrence intervals. Unlike the distributions derived from the zero-order model, these distributions did not differ significantly from the distributions observed in the training sets (one-tailed paired t-tests vs training set: zero-order, p<0.001; first-order p = 0.66; second-order p = 0.99), suggesting that even models that condition transition probabilities on a short preceding sequence can recover apparent longer-term structure in song sequences (Fig 2).

### Fisher’s exact tests for higher-order dependencies

The crucial difference between a first-order Markov process and a second-order (or higher) Markov process is that the second-order Markov process can contain non-adjacent dependencies between phrases [8]. In a sequence generated by a first-order process, observed transition probabilities depend solely upon the identity of the ultimate phrase type, while in a sequence generated by a second-order process, transition probabilities may be influenced by the identity of the penultimate phrase type.

**Fig 3:**
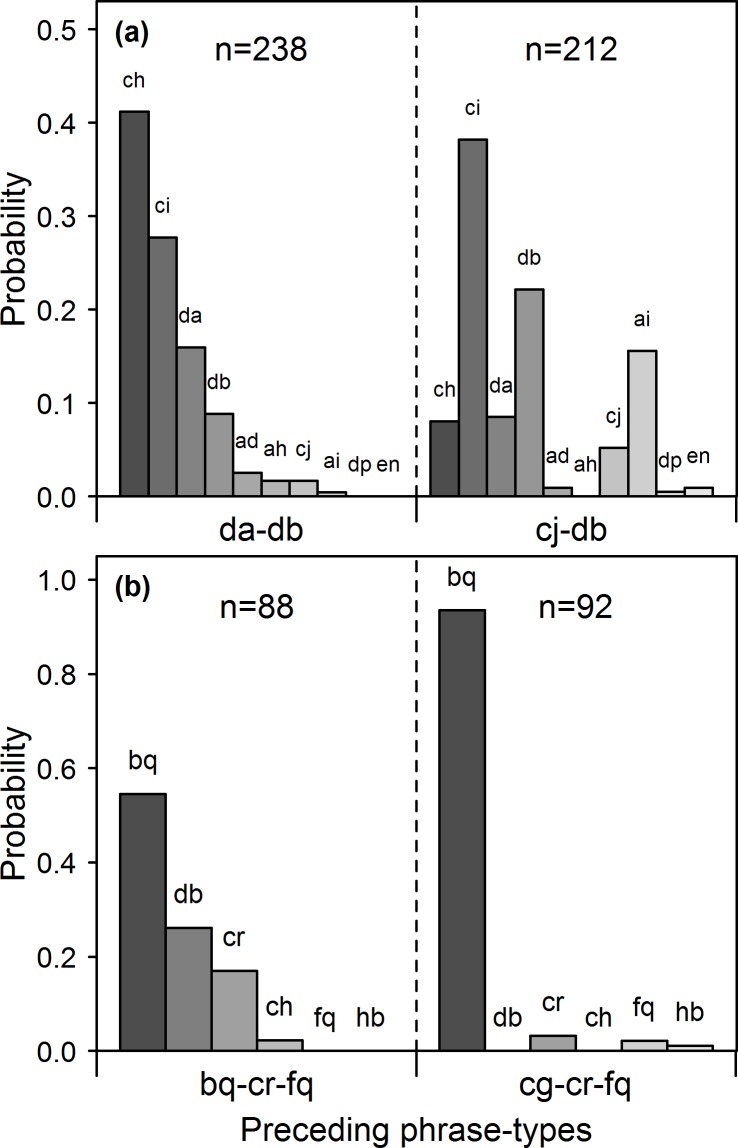
**A representation of second-order (a) and third-order (b) dependencies in the songs of the bird ‘AGBk’.** Bars represent the probability of observing a given phrase type given the identity of the preceding phrase types indicated below each graph. **a)** ultimate phrase type was *db*, and the probability distribution of upcoming phrase types changed depending on whether the penultimate phrase type was *da* or *cj* (Chi-square test for independence, *Χ*^*2*^(9, N = 450) = 116.94, p<0.0001). **b)** penultimate and ultimate phrase types were *cr* and *fq* in both cases, and the probability distribution differed with the identity of the antepenultimate phrase type, whether *bq* or *cg* (Fisher’s exact test, p<0.0001). Sample sizes indicate the number of observations upon which the probabilities were conditioned.

To investigate the presence of higher-order dependencies, I used Fisher’s exact tests to assess whether the probability of upcoming phrase types was influenced by the identity of the phrase type two or three phrases prior (Fig 3; see Materials and Methods). I found evidence of at least second-order dependencies in the songs of every individual (Table 1). On average, 46 Fisher’s tests were conducted to investigate second-order dependencies in each individual’s song sequences, and an average of 7.6 (16%) of these comparisons met the Bonferroni-corrected threshold for significance (Table 1). Each individual’s songs were subjected to a larger number of Fisher’s tests for third-order dependencies (mean = 160 comparisons). On average, 1.2 (1%) of these comparisons were significant at the Bonferroni-corrected significance threshold, though six individuals showed no evidence of third-order dependencies (Table 1). To control for the possibility that higher-order dependencies may arise spuriously, I simulated each bird’s recording corpus using a first- and second-order Markov model (see Materials and Methods). Sequences simulated using a first-order Markov model did not show evidence of second-order dependencies at the Bonferroni-corrected significance level, nor did sequences generated using a second-order Markov model show evidence of third-order dependencies, indicating that the significant results for the observed sequence reflect a more complex underlying syntax and could not have arisen spuriously. There was a positive correlation between the proportion of Fisher’s tests for third-order dependencies that were significant and the total number of phrases in a bird’s song corpus (Pearson’s test, r = 0.63, p = 0.017), suggesting that insufficient sample sizes may have impeded the detection of higher-order dependencies in the songs of some individuals.

### Model likelihood

I assessed the likelihood that each model could have generated observed sequences, using backoff smoothing and Witten-Bell discounting to account for events that were not observed in the training set [27,28]. The best model was that for which the probability of generating the observed sequence was highest, reflected in a low negative log-likelihood value (see Materials and Methods). In addition to the three models used previously, I used forward selection to interpolate between the models (see Materials and Methods), allowing assessments of models with a combination of zero-order, first-order and second-order properties. From these models of intermediate complexity, a model was selected (hereafter referred to as the ‘interpolated model’) that had the lowest negative log-likelihood on the testing set. This model was then assessed alongside the three original models using both a train-test paradigm and a Leave-one-out cross-validation (LOOCV) paradigm (see Materials and Methods). The inclusion of the interpolated model was based on the fact that the three original models differed significantly in their complexity: the zero-order model contained a single state; the first-order model contained a few dozen; and the second-order contained several hundred observed states. It seems likely that the syntax of any species will not abide strictly to any one of these models, and that the true complexity will often be intermediate between them. The use of forward selection to identify an interpolated model permitted evaluation of hundreds of candidate models with varying numbers of states in a straightforward and principled manner.

**Fig 4:**
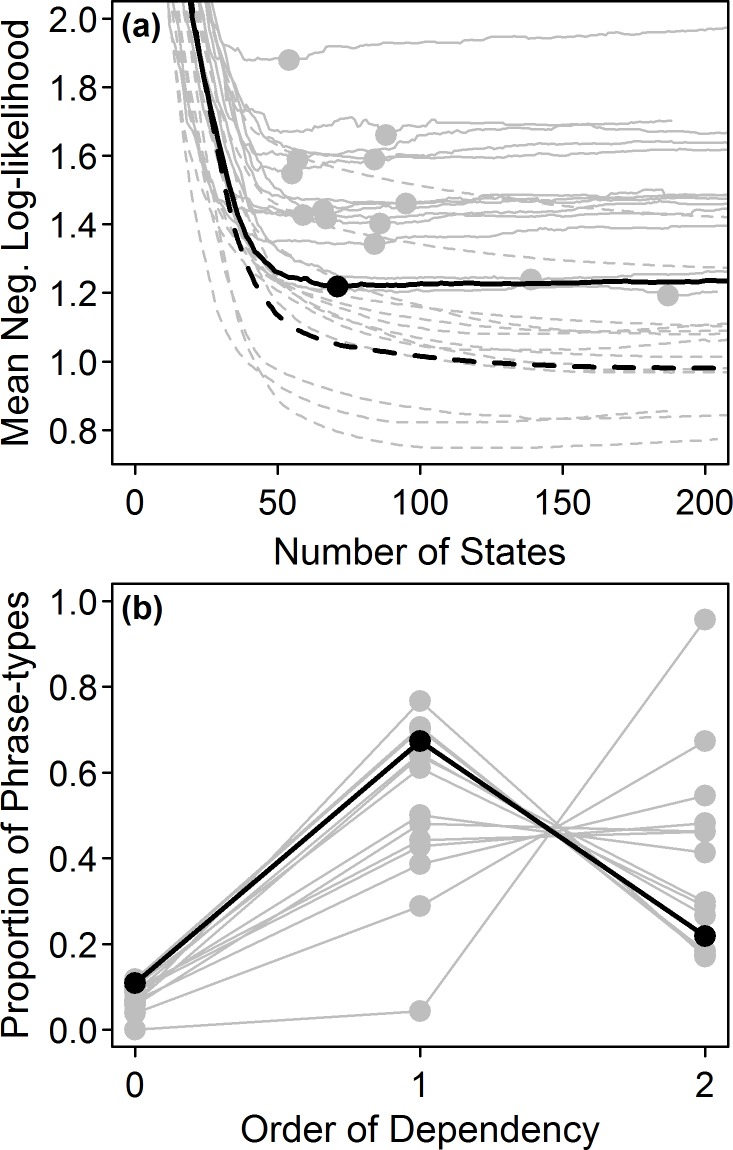
Illustration of the methodology and results of forward selection. Black lines show results for the individual ‘AGBk’; other individuals are shown in gray. **(a)** states were added to the model by selecting the state providing the largest improvement when applied to the training set (dotted line). The supplemented model was then tested on the testing set (solid line), and the method repeated until all second-order states had been added to the model. The best (interpolated) model was that which gave the minimum negative log-likelihood when applied to the testing set (solid circles). **(b)** structure of resulting interpolated models, showing the proportion of a bird’s repertoire exhibiting zero-order, first-order and second-order properties.

For all individuals, the interpolated model best fit the data under the train-test paradigm, though for two birds, the first-order model performed equally well (Table 2). Under the LOOCV paradigm, however, the interpolated model showed the best fit for all individuals (Table 2). The structure of the interpolated model differed between individuals, but always included a mixture of first- and second-order states (Fig 4). On average, 8% (range: 0–12%) of phrase types were grouped together within the zero-order category, suggesting that they did not show distinct first-order properties, or at least that their properties were not very different from one other. Fifty-two percent (range: 4–76%) of phrase types showed first-order properties, and 40% (range: 17–96%) showed evidence of second-order relationships. The interpolated Markov model included an average of 83 states, 66% more than the number of states in the first-order model, yet 67% fewer than the number of states in the second-order model.

**Table 2:** Results from likelihood and predictability analyses. L-0, L-1, L-2, and L-Int represent the average per-phrase negative log-likelihood under the zero-order, first-order, second-order, and interpolated Markov models, respectively. P-0, P-1, P-2 and P-Int indicated the prediction accuracy for the same models. Bolded values highlight the most likely model and the model with the best ability to predict upcoming phrases for each individual under the two evaluation paradigms.

Individual	Method	Sample size*	Likelihood				Prediction Accuracy (%)			
			L-0	L-1	L-2	L-Int	P-0	P-1	P-2	P-Int
AGBk	Train-test	5170	3.67	1.27	1.25	**1.22**	7.9	58.6	61.1	**61.6**
	LOOCV	10032	3.69	1.23	1.18	**1.16**	5.9	58.7	**63.2**	62.4
AGO	Train-test	2414	3.68	1.34	1.28	**1.24**	12.7	57.2	**63.4**	62.7
	LOOCV	4288	3.63	1.30	1.23	**1.19**	13.4	56.8	63.8	**64.1**
AOBu	Train-test	3883	3.68	1.62	1.65	**1.59**	7.7	46.9	45.4	**47.5**
	LOOCV	6634	3.66	1.56	1.58	**1.52**	6.1	47.9	49.5	**50.1**
AYO	Train-test	721	3.34	1.44	1.45	**1.42**	17.1	56.9	55.9	**58.1**
	LOOCV	1187	3.50	1.53	1.61	**1.51**	12.9	56.1	55.3	**57.7**
BuRA	Train-test	2698	3.53	1.89	2.06	**1.88**	6.8	40.4	41.0	**42.6**
	LOOCV	4460	3.59	1.81	1.88	**1.78**	4.6	41.8	42.7	**43.8**
Gate	Train-test	915	3.85	1.60	1.76	**1.59**	5.1	58.6	60.3	**61.4**
	LOOCV	1396	3.54	1.49	1.58	**1.46**	4.7	58.1	59.7	**60.4**
GRA	Train-test	1986	3.82	1.47	1.52	**1.44**	13.0	53.2	56.0	**56.8**
	LOOCV	3562	3.80	1.38	1.37	**1.35**	10.3	56.5	**60.1**	59.3
Meadow	Train-test	781	3.90	1.69	1.72	**1.66**	3.1	**52.7**	51.8	50.3
	LOOCV	1290	3.66	1.37	1.42	**1.35**	7.4	62.4	60.0	**62.6**
ORA	Train-test	1252	3.56	1.29	1.21	**1.19**	12.3	59.7	**64.2**	64.1
	LOOCV	2056	3.65	1.19	1.11	**1.09**	10.2	63.4	68.9	**69.3**
RYA	Train-test	1578	3.64	**1.43**	1.55	**1.43**	7.0	59.5	61.9	**62.2**
	LOOCV	2820	3.64	1.29	1.35	**1.27**	7.4	64.5	63.7	**65.6**
Gully	Train-test	1195	3.71	1.58	1.68	**1.55**	6.3	54.4	55.1	**57.0**
	LOOCV	1742	3.72	1.54	1.59	**1.50**	6.1	54.4	56.0	**57.8**
WABk	Train-test	4714	3.85	1.48	1.53	**1.46**	8.2	52.4	54.6	**54.7**
	LOOCV	8302	3.61	1.35	1.40	**1.33**	13.1	54.1	**56.2**	55.4
YAW	Train-test	1710	3.39	**1.34**	1.43	**1.34**	14.2	**62.6**	61.9	**62.6**
	LOOCV	2955	3.48	1.37	1.44	**1.36**	11.8	60.1	60.4	**61.1**
YBuA	Train-test	1558	3.54	1.49	1.50	**1.40**	6.0	57.2	61.9	**62.8**
	LOOCV	2760	3.59	1.53	1.51	**1.43**	5.1	57.2	61.9	**62.2**

*Sample size for the train-test paradigm was the number of phrases in an individual’s training set, while for the LOOCV paradigm, the sample size was the number of phrases in an individual’s total recording corpus minus the average number of phrases in each recording.

### Predictability of sequences

**Fig 5:**
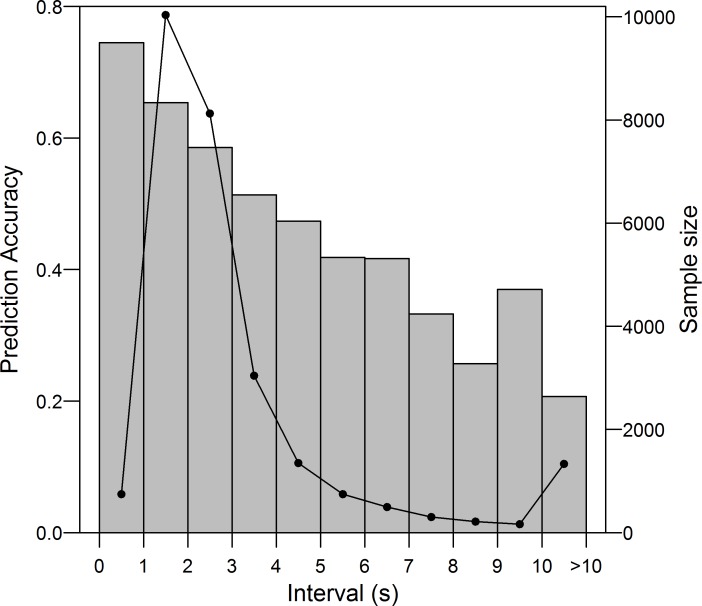
The relationship between the predictability of upcoming phrases and singing rate of Cassin’s Vireos (gray bars). Predictions were derived from the ‘interpolated’ Markov model using the train-test paradigm. Sample sizes for each interval are shown (black circles).

I examined the predictability of sequences using the four models used above. At each point in a sequence, the model returned the most likely phrase type based on the immediately preceding sequence (see Materials and methods). The zero-order model performed poorly for all individuals under the train-test paradigm, predicting upcoming phrase identity with an accuracy of 8.9%, while the first-order, second-order and interpolated models gave prediction accuracies of 54.2%, 56.1%, and 56.8%, respectively (Table 2). Under the LOOCV paradigm, the zero-order, first-order, second-order and interpolated models predicted upcoming phrase types with 8.5%, 55.3%, 57.9% and 58.2% accuracy, respectively (Table 2). A repeated measures ANOVA showed significant differences in the accuracy of the latter three models under the train-test paradigm (F(2,26) = 11.74, p<0.001) and the LOOCV paradigm (F(2,26) = 14.57, p<0.001). Under both paradigms, pairwise t-tests with Bonferroni-adjusted p-values determined that the first-order model gave significantly lower prediction accuracy than either the second-order or interpolated model (Train-test: first order vs second order p = 0.0496; first order vs interpolated p = 0.003. LOOCV: first order vs second order p = 0.034; first order vs interpolated p<0.001), but that the latter two models did not differ significantly in their prediction accuracies (both paradigms: p = 0.08). Though the magnitudes of these differences are small, this is because the first-order, second-order and interpolated models made identical predictions for 76% of phrases tested. Considering only instances where at least one model differed from the other two under the LOOCV paradigm (n = 13492 instances), the first-order, second-order and interpolated models had accuracies of 28.0%, 39.1%, and 40.3%, respectively, accounting for the differences in overall accuracy between the three models. Prediction accuracy depended strongly upon the length of the time interval across which the prediction was taking place: predictions across intervals shorter than two seconds, representing a high song output, were more than 65% accurate, declining to less than 25% for intervals longer than ten seconds (Fig 5).

### Time homogeneity

An assumption central to Markov models is that the process is time homogeneous, meaning that the transition probabilities do not change over time. This requirement is also critical to any attempt to model bird song syntax, as an explicit model of any bird’s syntax would be difficult to ascertain if the nature of the syntax were to change from one moment to the next, or from one month to the next. These data were particularly appropriate for investigating the time homogeneity of the songs because recordings were collected throughout the breeding season and, for some individuals, over multiple years.

**Fig 6:**
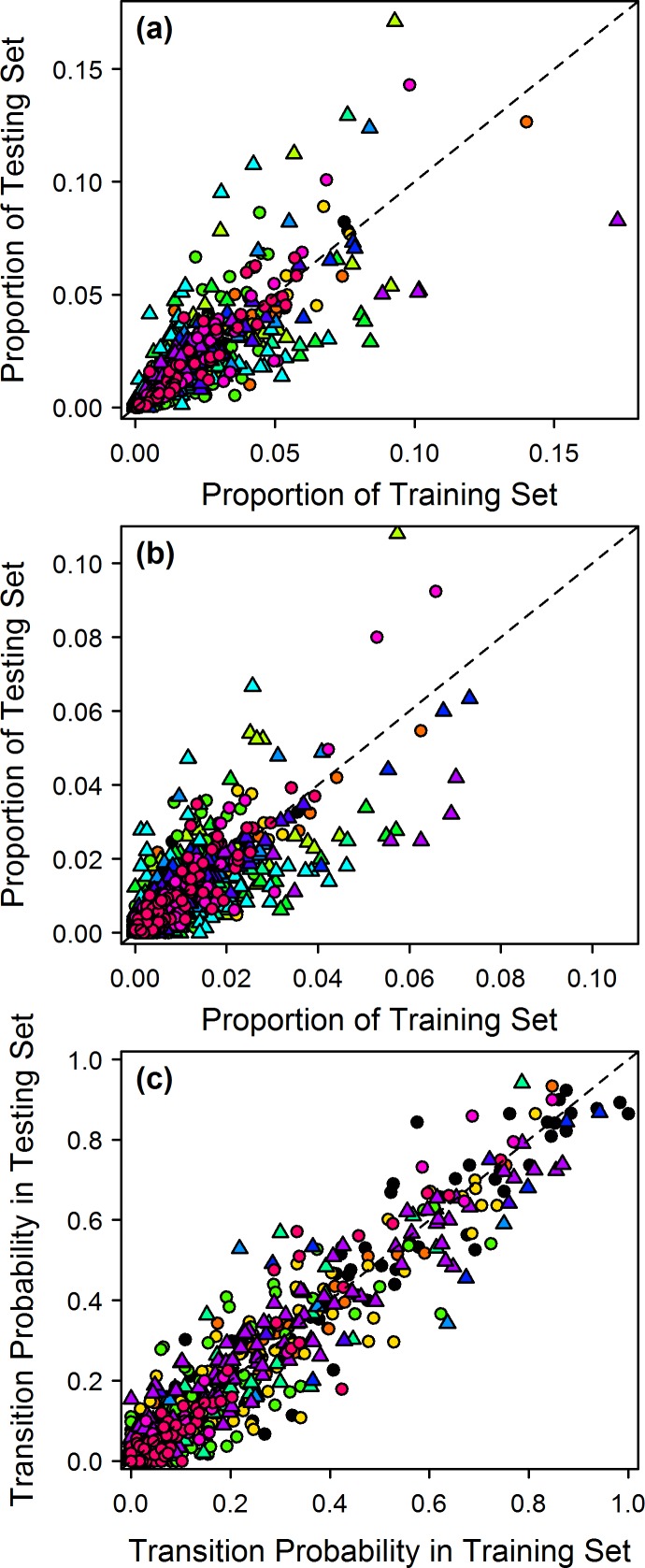
Phrase-type use, bigram occurrence, and transition probabilities observed in training sets and corresponding testing sets. Each point represents a phrase type **(a)**, bigram **(b)**, or transition probability **(c)** in a bird’s repertoire, and different individuals are represented by different colors. Individuals recorded in both 2013 and 2014 (n = 6 individuals) are denoted by circles, while those recorded in only a single year are denoted by triangles. Transition probabilities **(c)** were only calculated for probabilities conditioned upon at least 50 observations. Dotted lines have the equation y = x.

First, I observed that phrase-type use and transition probabilities appeared to be largely conserved between the training sets and testing sets used above. Phrase types that were common in a training set were also common in the corresponding testing set for each individual (Fig 6A, Pearson’s test, r(710) = 0.80, p<0.001). The same held true for bigrams (Fig 6B, Pearson’s test, r(4374) = 0.83, p<0.001), suggesting that this may hold for simple syntactic patterns as well as individual phrase types. Furthermore, first-order transition probabilities showed a strong correlation when considering only probabilities that were conditioned on a large number of observations (>50) in both the training and testing sets (Fig 6C, Pearson’s test, r(1534) = 0.96, p<0.001). Correlations between the training sets and testing sets were not perfect, however, raising the question of whether the disparity between the two sets was a result of gradual drift in repertoire use or syntax over time, or simply represented an artifact of the finite sample sizes of the two datasets. To assess this, I simulated recording corpora for each bird using second-order Markov models, which are, by definition, time homogeneous, and which above were shown to largely capture the syntactic structure of the songs. I then compared the L_1_-distances of the N-gram distributions for the 100 simulated training and testing sets to the values for the observed data (see Materials and Methods). The L_1_-distances are equivalent to the sum of the residuals around the line of equality (y = x) in Fig 6A (N = 1) and 6B (N = 2).

**Fig 7:**
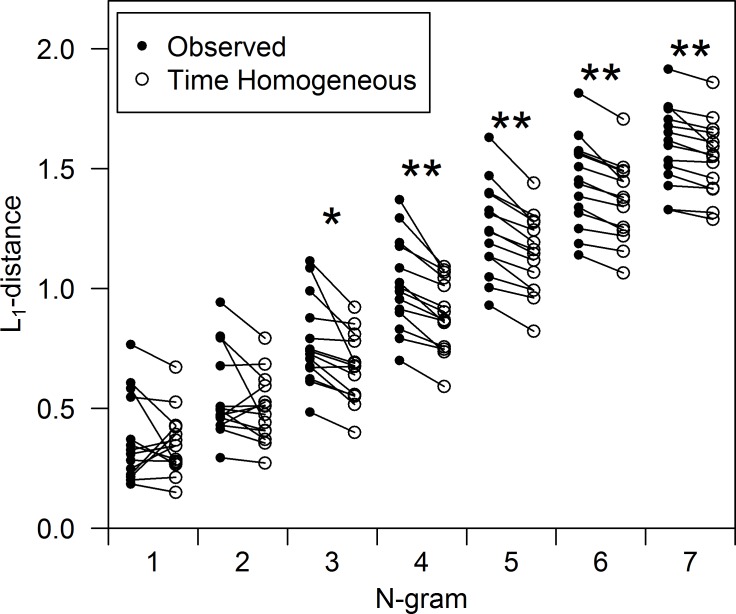
Comparison of L_1_-distances observed (closed circles) to the L_1_-distances expected under a second-order Markov model, which has the property of being time homogeneous (open circles). ‘Observed’ points were calculated by comparing the training set with the testing set for each individual, while the ‘Time Homogeneous’ points are average L_1_-distances from 100 simulated training and testing sets for each individual. Significant differences are denoted with asterisks (* = p<0.05, ** = p<0.01).

I found some evidence that the songs of the species were not completely time homogeneous, although the magnitude of the change in syntax did not appear great. At N = 1 and N = 2, the observed L_1_-distances were not different from those expected under a time-homogeneous second-order Markov process (Fig 7; p = 0.56 for N = 1; p = 0.14 for N = 2), indicating that the variability in Fig 6A and 6B are within the range expected under a time-homogeneous model. At higher values of N, however, the observed L_1_-distances were significantly larger than those expected under a time-homogeneous model (Fig 7; p<0.05 at N = 3; p<0.01 at N≥4), indicating that differences between the training and testing sets in these metrics were larger than expected under strict time homogeneity.

## Discussion

### Syntactic complexity

These results illustrate that the songs of Cassin’s Vireos are too complex to be modeled with either a zero-order or first-order Markov model. This conclusion was borne out by the sequence simulations, the Fisher’s exact tests, and the model likelihood analysis, all of which showed evidence for non-adjacent dependencies in the songs. Though sequences generated by a second-order Markov model appeared to more closely approximate the singing style of the species (Fig 2), the superior fit of the less complex interpolated Markov model in the likelihood analysis suggests that the second-order Markov models may contain unnecessary levels of complexity. The interpolated model did not, however, account for the third-order dependencies identified by the Fisher’s exact tests, so it is probable that some aspects of the syntax were not completely captured by that model.

An important consideration in the interpretation of these results is the influence of sample size on the detection of additional levels of complexity. That there was a significant positive relationship between individual sample sizes and the proportion of third-order dependencies suggests that sample sizes may constrain any attempt at inferring true levels of syntactic complexity in animal vocalizations; although sample sizes were in the thousands in this study, it is possible that with additional recordings, all individuals may have shown evidence of third-order or even higher-order dependencies. It may therefore be improper to draw strong conclusions regarding the upper bounds of syntactic complexity based on a finite recording sample. Attempts to rule out lower levels of complexity, however, are not faced with the same concerns. A conservative conclusion in light of this is that the rules underlying the syntax of Cassin’s Vireo songs are, at a minimum, intermediate in complexity between those that can be modeled by a first-order and a second-order Markov model, but that higher levels of complexity cannot be ruled out.

Regardless of the exact level of complexity of the songs, it appears that the syntax is governed by relatively simple rules and that transition probabilities are conditioned on the most recent phrase or phrases. Patterns that manifest themselves over longer time-scales, such as the tendency towards low recurrence intervals [25], appear to be emergent properties of these rules.

The patterns of complexity observed here are not unlike those observed in other species that have been the subject of thorough analyses of syntax. In Bengalese Finch, for example, Katahira et al. [15] identified second-order dependencies, and Jin and Kozhevnikov [16] proposed that a Partially-observable Markov Model (POMMA) best describes the species’ song sequences. Canaries have been shown to employ up to sixth-order dependencies, describable by a Prediction Suffix Tree (PST) [17]. Common to both the POMMA and PST models is the ability to model higher-order dependencies, such that each phrase type can be associated with more than one model state. This many-to-one mapping between model states and observable output is also a characteristic of second-order Markov models, and is a key feature of the interpolated Markov model that provided the best fit to the Cassin’s Vireo sequences.

The similarity in syntactical complexity between these species is particularly noteworthy given the distant evolutionary relationship between Cassin’s Vireo, members of the clade Corvoidea, and Bengalese Finch and Canary, of the clade Passerida–clades that have been separated by more than 25 million years of evolution [29,30]. This apparent convergence towards similar syntactic properties suggests this pattern may be a widespread phenomenon in songbirds. Previous authors have posited that many-to-one mapping between internal states and vocal output may be a natural consequence of song development–either through multiple memorization of the same phrase type under different syntactic contexts [31,32], or through many-to-one neuronal projections between disparate song nuclei in the brain [32,33]. Another possibility is that these similarities reflect an upper limit to the complexity of syntactic rules that can be stored in, and produced by, the brains of songbirds. Such a limit has been proposed by previous authors that have noted the relative simplicity of bird song syntax when compared with that of human language, with particular emphasis on the apparent lack of recursion in bird song [18–20]. This hypothesis is difficult to evaluate, however, without a more complete understanding of the evolutionary costs and benefits associated with complex syntax. Gentner and Hulse [34] showed that European Starlings produce sequences that are well approximated by a second-order Markov model, and also showed that individuals of that species could differentiate sequences generated by first- and second-order Markov models, but not those generated by second- and third-order models. They proposed that one factor constraining syntax may be the ability of receivers to process additional complexity above some limit, which correspondingly restricts the benefits of evolving more complex song output. Alternatively, song syntax may be limited by neural constraints on the part of the sender, though the neural demands associated with complex song syntax are still not well understood [35]. Future research should attempt to classify the complexity of syntax in a wider array of species, and to identify the role of complex syntax in communication, with the goal of understanding the evolution of this trait and explaining similarities and differences in singing strategies in songbirds.

### Predictability of sequences

These results showed that, given sufficient knowledge of a bird’s syntax, the identity of upcoming phrase types could be estimated with more than 54% accuracy, and more than 65% accuracy when song output was high (Fig 5). Aligning with the likelihood analyses and simulations, the model with the highest accuracy was the interpolated model, though the differences in performance between this model and the first- and second-order models were less than three percentage points. It is doubtful that the differences between these three models are of biological significance, as the results show that a bird attempting to predict the identity of upcoming phrases would do nearly as well having heard a single phrase as if they had heard two or more. More likely to be important from a biological perspective are the high levels of overall determinism in the sequences. During territorial disputes, many species of birds are known to engage in song matching, in which the songs of one bird will be immediately repeated by the other. This behavior has been proposed to have a role in the evolution of song repertoires [3] and geographic dialects [36]. In most species that have been the focus of song matching research, however, birds sing with eventual variety (e.g. Song Sparrows [37,38]), repeating each song type many times consecutively. In such cases, the predictability of songs is high: the next song type in a sequence is likely to be the same as the previous. To match a rival’s songs, a male need only repeat the most recently heard song type.

In contrast, species that sing with immediate variety, constantly switching between song types, are faced with a greater challenge when attempting to match songs, especially when the intervals between songs are short, as in the songs of Cassin’s Vireo. The ability to predict upcoming events could conceivably facilitate song matching by allowing individuals to anticipate upcoming phrases rather than react to them. Counter-singing interactions between rival males are common across territorial boundaries in this species, and I have observed many interactions that appear to contain significant amounts of phrase-type matching. The increased predictability of songs when singing at high rates may play a role in mediating territorial disputes, which often involve rapid exchange of song. Further investigations into the specific role of syntactic rules during song matching interactions are ongoing and promise to yield further insight into the functional importance of song syntax in this species.

### Non-Markovian properties

#### Time homogeneity

The songs of Cassin’s Vireo appear to be nearly time homogeneous, but not completely so. The observed differences between the training and testing sequences in the 1-gram and 2-gram distributions were within the range of variation expected under a time-homogeneous second-order Markov model, whereas the higher order N-grams exhibited larger differences than would be expected if the syntax were time homogeneous (Fig 7). In other words, the variability in Fig 6A and 6B is within the range expected under a time-homogeneous model, but a similar plot of higher N-grams would show somewhat more variability than expected.

These differences could have resulted from a few factors. First, it is possible that the delivery of songs depends upon external variables, such as the presence of females [39], rivals [40], or predators [41]. As an example of this, I have observed that Cassin’s Vireos sing particular phrase types near the nest as the female incubates, an observation that has also been noted in Yellow-throated Vireos [42] and is the focus of ongoing research. It follows, then, that recordings made during nesting may be enriched for transitions and N-grams containing these particular phrase types, and impoverished for others. Second, as discussed above, Cassin’s Vireos regularly engage in phrase-type matching interactions with neighbors, implying yet another influence of social context on their singing behavior.

Both of these potential social influences on song occur over short time scales, on the order of seconds or minutes. There does not appear to be evidence that the syntax of the species drifts over long periods, as this would likely have led to much larger differences between the training set and testing set, especially in the individuals that were recorded in two breeding seasons (Fig 6 and Fig 7). The observations presented here align with the findings of Warren et al. [43], who showed that Bengalese Finch syntax is stable over time, but can change in response to external factors. They showed that transition probabilities could be altered using aversive stimuli, but that probabilities returned to baseline values following the experiment, suggesting that although birds can alter their syntax over short time-scales, their preferred syntax remains consistent throughout their adult life.

The relatively stable nature of the repertoire and syntax over time aligns with current understandings of bird song development in which many species of birds undergo a sensitive period of song acquisition, before their songs crystallize and remain relatively unchanged throughout their adult life [44], with modifications in adulthood being minor adjustments rather than gross reorganizations of song syntax or repertoire composition [45].

#### Repetitions of phrase types

Previous attempts to model the syntax of birds have often modeled separately the transitions between vocal units and the patterns of repetition of units [16,17,32,46]. For many species, this is critical because the repeat distributions are clearly non-Markovian [47], and appear to be governed by processes distinct from those governing transitions between phrase types. I did not model these two processes separately, justified by the observation that repetitions of phrase types are uncommon in this species, accounting for less than 7% of consecutive phrases. Triplets of the same phrase type were even rarer, accounting for less than 2% of consecutive phrases, while quadruplets accounted for 1% of the observed song corpus. A second-order Markov model captures the probabilities of repetitions up to length three, so the models evaluated here likely approximated the distribution of repeated phrases reasonably well. I did, however, occasionally observe highly anomalous singing behavior characterized by long bouts of repetitions. In one case, an individual repeated the same phrase type 38 times, a sequence that would be infinitesimally improbable under any of the models examined here. The current models may be improved somewhat by treating repetitions differently from transitions between phrase types [16], but given the rarity of these events in this species, it is likely that any improvements attained in doing so would be minor.

These results illustrate a moderate level of complexity in the syntax of Cassin’s Vireo songs. The sequences examined show abundant evidence of second-order dependencies, and hint towards a level of complexity somewhere between that of a first-order and second-order Markov model, though there was also some evidence for higher levels of complexity. It is clear that there are ample opportunities for future research, especially regarding the functional significance of a highly structured syntax during song matching and other social contexts.

## Materials and Methods

### Recording techniques and individual identification

All field research was approved by the Institutional Animal Care and Use Committee at the University of California, Los Angeles (protocol number ARC # 2013-041-01). Banding was carried out under federal bird banding permit #23809. I collected recordings between April 25 and June 28, 2013, and between May 5 and June 25, 2014 at a field site on private land in the foothills of the Sierra Nevada Mountains in California, USA (10 S 706584 4262742, datum WGS 84). All recordings were made with a Marantz PMD 661 solid state digital recording unit and a Sennheiser MKH20-P48 microphone with a Telinga parabolic reflector, and recordings were stored as 16 bit WAV files with a sampling rate of 44.1 kHz. I recorded birds opportunistically by approaching a known breeding territory and recording the singing male until he either stopped singing or flew too far away to be recorded. In 2013, males were identified based on their association with known breeding territories, and identifications were then confirmed from recordings based on the observation that birds possess individually distinctive repertoires that are organized into diagnostic sequences [48,49]. In May 2014, males were captured and marked with unique colored leg bands, which helped identify the birds during subsequent recordings. Individuals are here referred to by a code representing their color bands, except in three birds that were not captured, referred to as ‘Gate’, ‘Meadow’ and ‘Gully’.

### Recording annotation

Recording annotation methods were the same as those described in Hedley [25], and many of the recordings analyzed here were also included in that study. All recording annotation was completed using the program Praat [50]. I visually inspected the spectrogram of each recording, and marked the boundaries of each phrase with approximately +/- 0.01s accuracy. Each phrase was classified as one of 126 phrase types that have been observed in the study population, based on distinctive acoustic characteristics that are readily visible on the spectrogram; phrase types were denoted with unique two-letter codes (S1 File). A spectrogram showing four phrases with their corresponding annotations is shown in Fig 1.

Although the identification of phrase types is subjective, Tan et al. [51,52] have shown that the categories used here were objectively identifiable by computer algorithms, and a comparison between visual inspection and algorithmic classification showed that the two methods agreed on more than 99% of occasions when tested on recordings with high signal-to-noise ratios [25]. Similarly, a comparison of annotations made by two observers on 100 phrases from each of the fourteen individuals in this study showed that the two observers assigned the same phrase type label for 99% of phrases (1386/1400 phrases). The visual inspection method from a single observer was used for all annotations due to potential concerns about noise-robustness of the classification algorithm and the prohibitive time and personnel requirements of using multiple observers.

In accordance with the methods of Hedley [25], I discarded all recordings containing fewer than 50 phrases, which amounted to four percent of the total phrases in the original recordings. The resulting annotated dataset comprised 221 recordings containing 57377 phrases from fourteen individuals (mean = 4098, range = 1335–10336 phrases per individual). All sound recordings are available on Figshare (10.6084/m9.figshare.3081814), along with corresponding textgrid files containing phrase annotations, for use with Praat [50]. Metadata for each recording are provided in S1 Table.

### Simulations

#### Model parametrization

Each bird’s recording corpus was divided into a training set and a testing set by concatenating the sequence from all recordings of a given individual in chronological order and identifying the midpoint of the sequence. The recording containing this phrase was placed in the training set along with all preceding recordings, while the remaining recordings were held out as the testing set. As such, each recording was assigned to either the training set or the testing set, never split in two. This procedure for dividing a bird’s recording corpus in is referred to as the ‘train-test paradigm’ throughout this study.

The zero-order Markov model was the simplest model investigated. In this model, the probability of observing a given phrase type in a sequence is independent of the identity of the preceding phrases, and is proportional to its frequency of occurrence in the training data. The probability of each phrase type was calculated as

$$P_{i}=n_{i}/N$$

Where *n*_*i*_ is the number of times phrase type *i* was observed in the training set, and *N* was the total number of phrases in the training set.

The model with intermediate complexity was a first-order Markov model, in which the probability of observing a given phrase type depends on the identity of the immediately preceding phrase. This model can be visualized as a *C*x*C* matrix, where each row and column represents a phrase type in the bird’s repertoire. The rows represent the identity of the preceding phrase, while the columns represent the identity of the subsequent phrase. The probability of transitioning from phrase type *i* to phrase type *j* is estimated as

$$P_{ij}=P(j|i)=n_{ij}/n_{i•}$$

Where *n*_*ij*_ is the number of times phrase type *j* followed phrase type *i* in the training sequence, and *n*_*i*•_ is the total number of bigrams observed that began with phrase type *i*. The value of *n*_*i*_• was sometimes slightly less than *n*_*i*_ when phrase type *i* was the terminal phrase of a recording. Typically, however, these two values were equal.

The most complex model examined here was a second-order Markov model, in which the probability of observing a given phrase type depends on the identity of the two preceding phrases. This model can be represented as a matrix containing *C*^2^ rows and *C* columns, where each contains a unique pair of the *C* phrase types in the training set. Transition probabilities are estimated as

$$P_{ijk}=P(k|ij)=n_{ijk}/n_{ij•}$$

Where *n*_*ijk*_ is the number of times the trigram *ijk* was observed in the training set, and *n*_ij•_ is the total number of trigrams beginning with the bigram *ij*. Considerations of sample sizes precluded looking at higher order Markov processes.

#### Sequence simulations

Simulations were designed such that the only difference between a simulated dataset and the training data was the mode of syntax generation: since each training set was comprised of multiple recordings containing varying numbers of phrases, each simulation contained the same number of simulated recordings with the same numbers of phrases. Furthermore, the first phrase type (or two phrase types, in the case of the second-order model) in each simulated recording was made to be identical to those observed in the training set. Unlike subsequent analyses, transition matrices were not smoothed prior to simulations (see below). One ‘simulation’ therefore was equivalent in file structure, length and initial state to the training set from which it was parameterized. Each individual’s training set was simulated 1000 times using the stochastic random sampling function *sample* in R [53].

#### Model assessment

The resulting simulations were compared with the testing set in two ways. First, the N-gram distribution of the simulated data was compared to that of the testing data for N-grams between one and seven. The N-gram distribution is the relative frequency of all unigrams (for N = 1), bigrams (N = 2), trigrams (N = 3), etc. in the sequence. The 1-gram distribution was the relative frequency of each phrase type in the sequence, calculated by dividing the number of observed instances of each phrase type by the total length of the sequence. The 2-gram distribution was calculated similarly, by dividing the number of observed instances of each bigram by the total number of bigrams in the sequence. Higher-order N-gram distributions were calculated in a similar fashion. Because each N-gram distribution contained relative frequencies, the entries of each N-gram distribution summed to one. The lack of smoothing prior to the sequence simulations constrained the N-grams that could be observed generated at low values of N: the zero-order model was constrained to the 1-grams in the training set; the first-order model was constrained to the 2-grams in the training set; and the second-order model was constrained to the 3-grams in the training set. Each of these models could generate a greater variety of N-grams at higher values of N, provided they were comprised of lower N-grams present in the training set. Differences between simulated sequences and observed sequences were therefore expected to be most prominent at higher values of N.

Comparison of N-gram distributions was conducted by taking the L_1_-distance between the two distributions, where *L*_1_ − *distance* = ∑_*i*_|*x*_*i*_ − *y*_*i*_|, *x*_*i*_ is the frequency of occurrence of a particular N-gram in one dataset, and *y*_*i*_ is the frequency of occurrence of that same N-gram in the second dataset. Because this approach measures the difference between two distributions that each sum to one, this measure of similarity can take values from 0 to 2. Two distributions with an L_1_-distance of 0 are identical, those with a distance of 1 show exactly 50% concordance in their probability distributions, and those with a value of 2 do not overlap at all; the L_1_-distance therefore corresponds with the amount of probability mass that must be redistributed to render the two distributions identical. This same analysis was conducted using the sum of squared differences, which more heavily weights outliers, and the results were qualitatively similar; L_1_-distances were used for their ease of interpretation.

The second way I compared simulated sequences with the observed data was through the distribution of recurrence intervals. The recurrence interval was defined as the number of intervening phrases between two occurrences of the same phrase type [54]. This characteristic was chosen because it evaluates sequential structure across the full length of each recording, and so complements the characteristics assessed by the N-gram distributions, which analyzed the structure across short chunks of song, up to seven phrases in length. Recurrence intervals have been shown to be diagnostic characteristics of bird song for various species. Some species sing with high recurrence intervals, reflecting a tendency to cycle through the repertoire (e.g. Marsh Wren [55], Western Meadowlark [12], Chaffinch [31]), while other species favor low recurrence intervals, presenting small subsets of their repertoires at a time (e.g. Rock Wren [54]). Suitable syntactic models should generate songs with species-typical recurrence intervals.

The songs of Cassin’s Vireos favor low recurrence intervals, meaning that after a phrase type is delivered once, it is likely to recur shortly thereafter in a sequence [25]. I calculated the observed distribution of recurrence intervals for each simulated sequence and for the training set and testing set, then compared the training set and simulated data to the testing set using the L_1_-distance in the same manner as above.

### Fisher’s tests for higher-order dependencies

To identify second-order dependencies, I calculated a second-order Markov transition matrix using the complete corpus of each bird’s songs. For each phrase type in a bird’s repertoire, I compared the probability distributions associated with two states, where the states differed in the identity of the penultimate phrase type but not in the ultimate phrase type. I used a Fisher’s exact test to determine whether the observed transition probabilities differed as a function of the identity of the penultimate phrase type while keeping the ultimate phrase type constant (Fig 3). The two states selected for comparison were the two containing the largest number of observations because, despite large overall sample sizes in this study, the number of observations contributing to the estimation of each transition probability was often small. Considerations of sample size are examined further in the discussion. In about 3% of comparisons, sample sizes were too large to calculate exact p-values using Fisher’s exact tests, so Chi-square tests for independence were used instead. I used Bonferroni corrections to control for multiple comparisons within each bird’s repertoire. The total number of comparisons was often less than the repertoire size because some phrase types were only observed to be associated with a single second-order state, making comparisons unfeasible for that phrase type.

As a point of comparison, I simulated a complete recording corpus for each bird under a first-order Markov model and conducted the same analysis on the simulated data, with the expectation that a sequence generated by a first-order Markov model would show little or no evidence of higher-order dependencies.

I conducted the same analysis to investigate third-order dependencies by calculating the third-order Markov matrix, and comparing states that had the same ultimate and penultimate phrase type, but differed in their antepenultimate (third most recent) phrase type. Similar to above, only the two states with the largest sample sizes for a given pair of ultimate and penultimate phrase types were compared. The total number of comparisons was somewhat less than the total number of bigrams observed in each bird’s recording corpus, because some bigrams were only ever preceded by a single antepenultimate phrase type and therefore could not be included in this analysis. Again, I simulated each bird’s corpus, this time using a second-order Markov model, and conducted the same analysis on the simulated sequence to rule out the possibility of spurious relationships.

### Model likelihood

#### Model evaluation

Another measure of the fit of a model to unseen data is the negative of the log-likelihood of a sequence given a particular model. Negative log-likelihood is measured by multiplying the probability of observing each phrase type at each point in the testing set, given the hypothesized model of sequence generation parametrized from the training set, then taking the negative of the natural logarithm.

The negative-log-likelihood of the sequence as a whole is given by

$$-lnL(sequence|model)=-ln\prod_{m}P(x_{m}|model)$$

Where *P*(*x*_*m*_|*model*) is the probability of observing phrase type *x* at position *m* given the model in question. Probabilities of each observation given each candidate model are given by *P*_*i*_, *P*_*ij*_, and *P*_*ijk*_ above for the zero-order, first-order and second-order models respectively.

#### Forward selection

The forward selection method used here was derived from the method outlined in James et al. [23], and shares similarities with the methods used by Markowitz et al. to construct Prediction Suffix Trees that were used to identify long range dependencies in Canary song [17]. Starting with the zero-order model, I evaluated all possible first-order states that could be added to the model, selecting the state that provided the greatest reduction in negative-log-likelihood when applied to the training set. The model was then evaluated on the testing set. Subsequently, first- or second-order states could be added to the model, with the constraint that a second-order state was only considered if its corresponding first-order state had already been included. The process concluded once all of the second-order states had been added to the model. The ‘interpolated model’ was that which had the lowest negative-log-likelihood when applied to the testing set, and could contain states with any combination of zero-order, first-order and second-order properties.

#### Evaluation paradigms

The fit of each model to the data was assessed in two ways: first, the model was parametrized on the training set and evaluated on the testing set according to the train-test paradigm described earlier. This method was used to select the interpolated Markov model. The second method used was leave-one-out cross-validation, which I refer to as the ‘LOOCV paradigm’. In this case, the model was iteratively parametrized on all but one recording from a bird’s corpus, and was evaluated on the held out file. This method acted as a check against the possibility of overfitting when using a single training set.

For each testing set, the first two phrases in each recording were not included in the calculation, since the second-order model requires knowledge of the two preceding phrase types. I divided the resulting likelihood value by the total length of the sequence to control for the different lengths of sequences in the recordings of each bird, such that the values presented represent the negative-log-likelihood averaged across each phrase in the sequence.

#### Smoothing

Some of the transitions observed in the testing set were not observed in the training set, and therefore had estimated probabilities of zero, making the likelihood calculation above meaningless. To overcome this issue, I used backoff smoothing to provide non-zero probabilities to each cell in the transition matrix. Backoff smoothing uses information from simpler models to estimate probabilities for unseen observations in a more complex model [28]. For example, if the transition *aa-ab-ac* was not observed in the training set, the algorithm uses the probability obtained from the first-order model for the transition *ab*-*ac* to estimate the probability. If this transition was also not observed, the algorithm ‘backs off’ further to the zero-order model, using the probability of the phrase type *ac* to provide a non-zero estimate. Observed probabilities must be lowered to account for the added weight to the unobserved transitions, and for this I used Witten-Bell discounting, which reduces each probability by the factor *N*/(*N* + *T*), where *N* is the number of observations upon which the probability was conditioned, and *T* is the number of different phrase types that were observed under the given condition [27]. The remaining weight, equal to *T*/(*N* + *T*), is redistributed amongst the unobserved phrase types according to the backoff algorithm described above.

### Predictability of sequences

To assess the ability of each model to predict upcoming phrase types, I used the transition matrices to predict the most likely upcoming phrase type based on recently observed sequences, and assessed the accuracy of each model based on the extent to which the predicted phrase types agreed with those observed in the data. The zero-order model always output the most common phrase type in the training set, independent of the preceding sequence, while the first- and second-order models predicted the most commonly observed phrase type given the most recent and two most recent phrases, respectively. The interpolated model used information from one or two preceding phrase types to make its prediction, depending on whether the preceding sequence pertained to a zero-order, first-order or second-order state in the model.

### Time homogeneity

Previous work assessing the time homogeneity of Markov processes has relied upon dividing observed sequences into multiple subsets and comparing transition matrices derived from each subset using Chi-square tests [56,57]. Such methods would not be suitable here, however, since the transition matrix for Cassin’s Vireo songs contained several thousand elements, many of which were rarely or never observed. Instead, I used simulations to evaluate the extent to which the differences between the observed training and testing sets deviated from the differences that would be expected under a time-homogeneous process.

I simulated each bird’s recording corpus 100 times using a second-order Markov model. In contrast to previous simulations, the entire recording corpus was used for model parametrization. Recording lengths, initial phrase types, and sample sizes of the original data were maintained as above, and the simulated corpus was subsequently divided into ‘simulated training’ and ‘simulated testing’ sets corresponding to the files in the training and testing sets in the train-test paradigm, such that the simulated and observed sets differed only in their means of generation–the simulated sets by a time-homogeneous second-order Markov process, the observed sets by the birds themselves. As in the earlier simulations, smoothing was not employed here.

For each individual at each N-gram value, I calculated the L_1_-distance between each of the 100 pairs of simulated training and simulated testing sets using the L_1_-distance. This resulted in a distribution of L_1_-distances that would be expected under a time-homogeneous second-order Markov model, with the expectation that a time-inhomogeneous process would show significantly larger L_1_-distances than the simulated data. To test for significance, I calculated the mean and standard deviation of the simulated L_1_-distances for each individual at each N-gram, and calculated corresponding z-scores for their observed L_1_-distances at that N-gram. I then conducted a one sample t-test to examine whether the observed z-scores differed significantly from zero, which would suggest a time-inhomogeneous process.

## Supporting Information

S1 File Classification key for phrase types. Songs delivered by Cassin’s Vireos were classifiable based on their appearance on spectrograms. Red horizontal lines are drawn at a frequency of 3kHz for comparison. In total, 126 phrase types were identified among the 14 individuals analyzed here. Each phrase type was given a unique two-letter code.
(PDF)

S1 Table Metadata for sound recordings. Information about the identity of the subject of each recording, and the number of phrases, duration, and recording time and date for each recording.
(XLSX)
