# Supplementary material for: Complexity, Predictability and Time Homogeneity of Syntax in the Songs of Cassin’s Vireo (Vireo cassinii)
Source: PLoS One. 2016 Apr 6;11(4):e0150822. doi: 10.1371/journal.pone.0150822 (PMC4822860; doi:10.1371/journal.pone.0150822)
Supplement: S1 File — Songs delivered by Cassin’s Vireos were classifiable based on their appearance on spectrograms. Red horizontal lines are drawn at a frequency of 3kHz for comparison. In total, 126 phrase types were identified among the 14 individuals analyzed here. Each phrase type was given a unique two-letter code. (PDF) [file pone.0150822.s001.pdf]

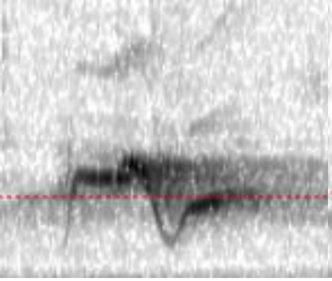
aa

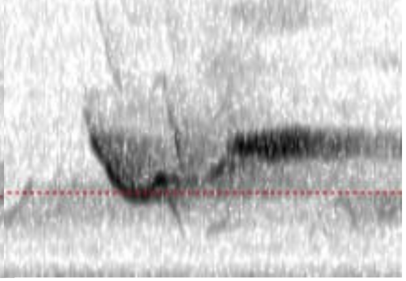
ab

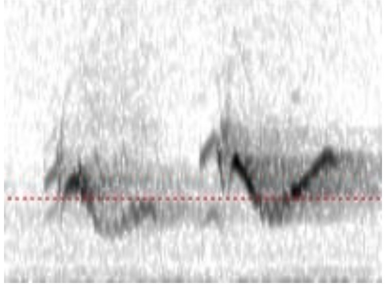
ac

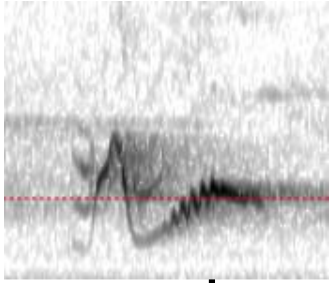
ad

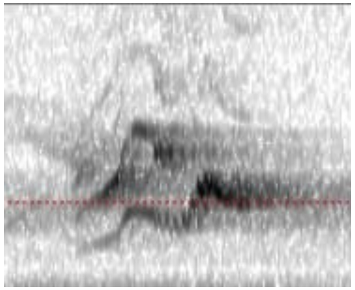
ae

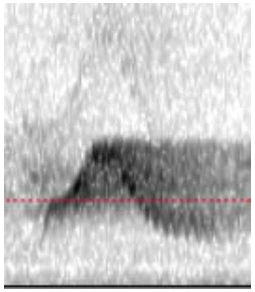
af

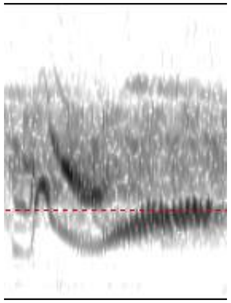
ag

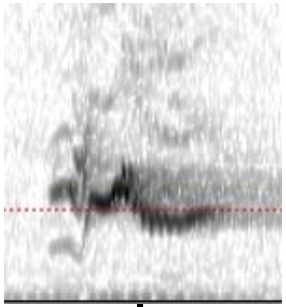
ah

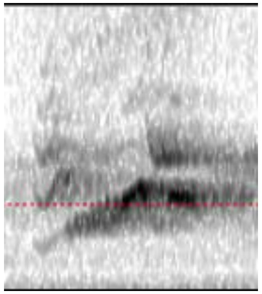
ai

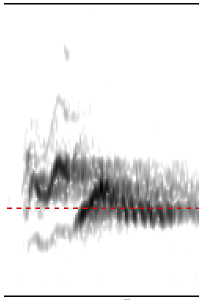
aj

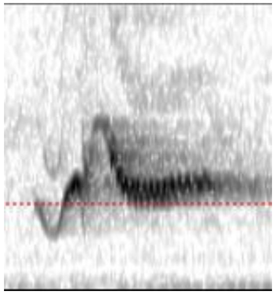
ak

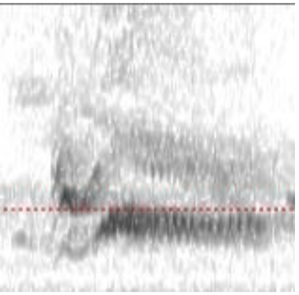
am

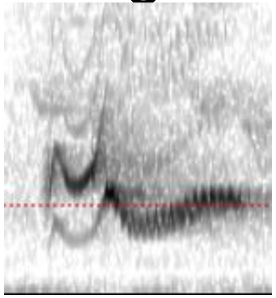
an

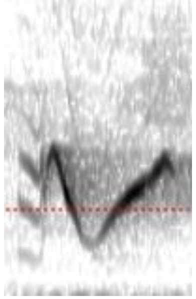
ao

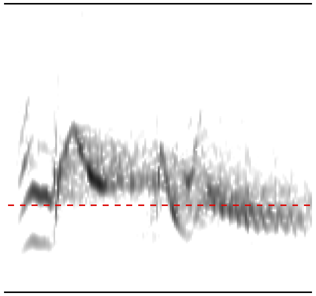
ap

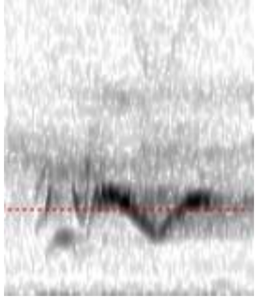
aq

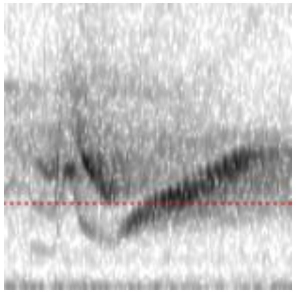
ar

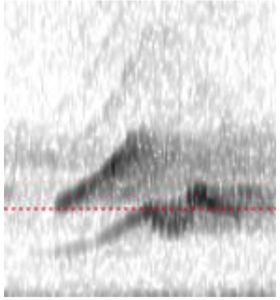
as

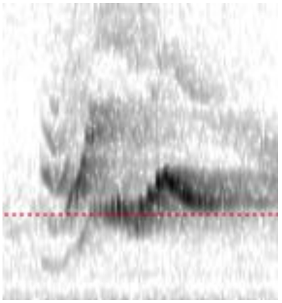
au

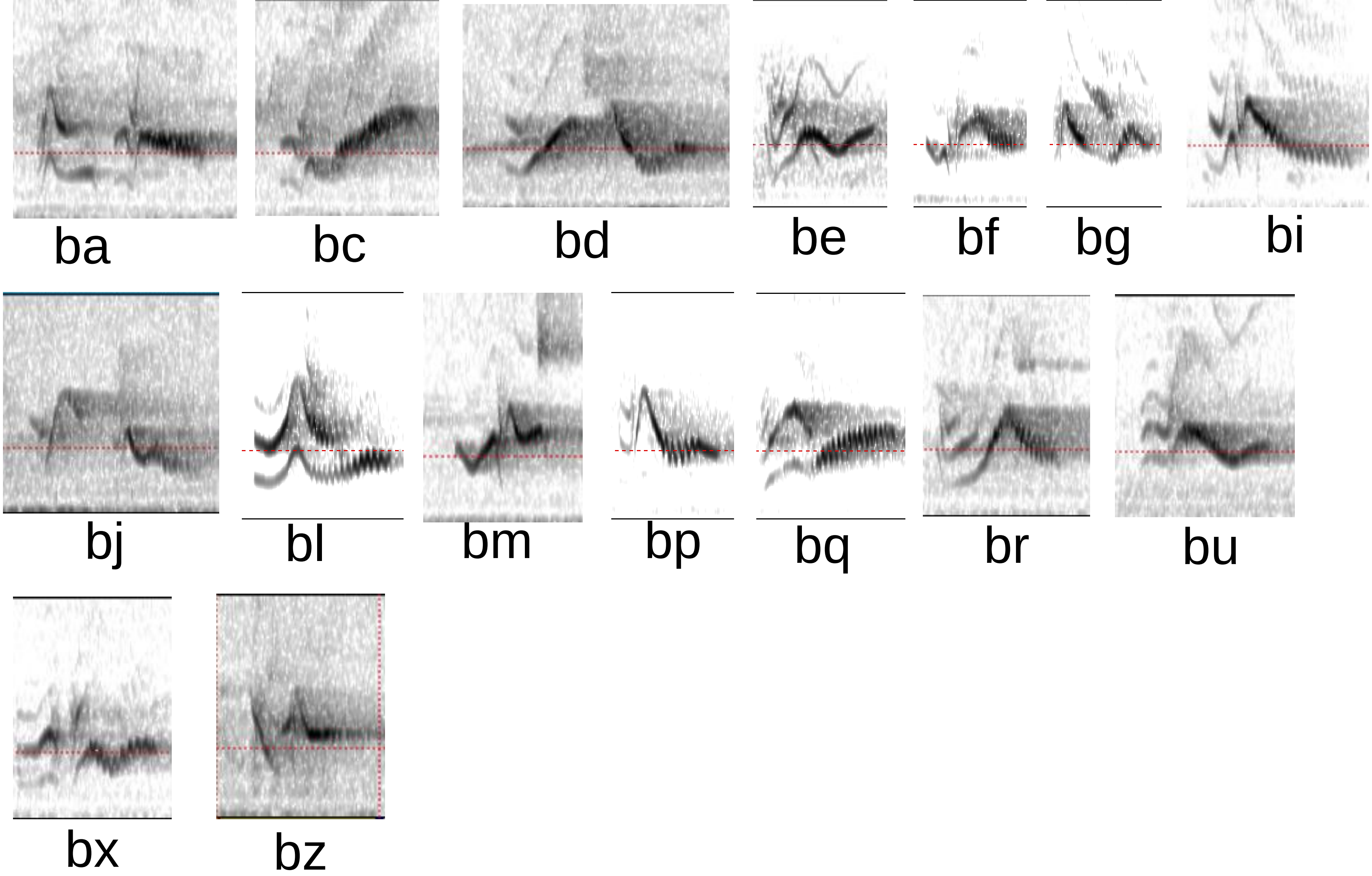
ba
bc
bd
be
bf
bg
bi
bj
bl
bm
bp
bq
br
bu
bx
bz

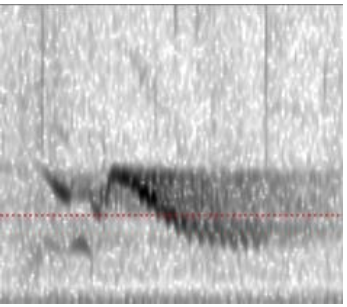

ca

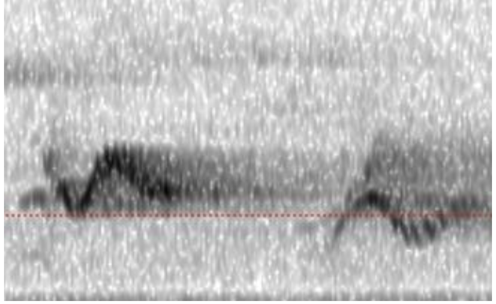

cb

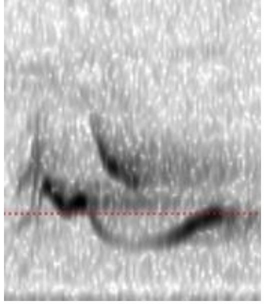

cc

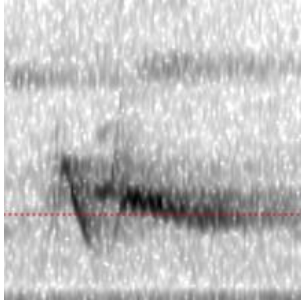

cd

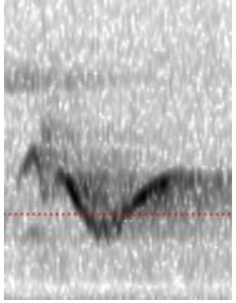

ce

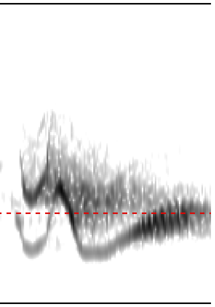

cg

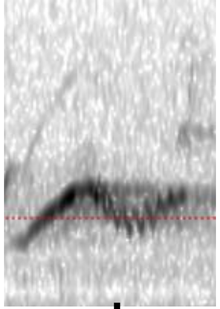

ch

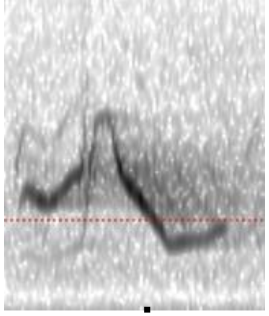

ci

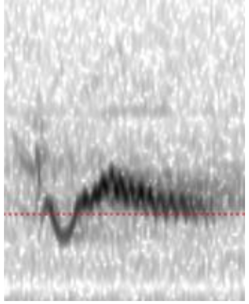

cj

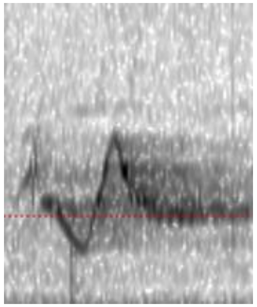

ck

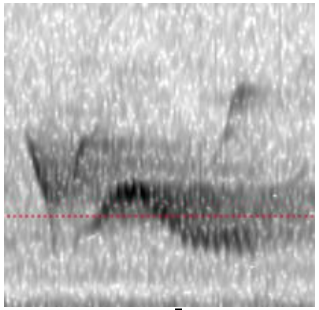

cl

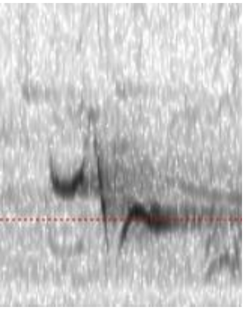

cm

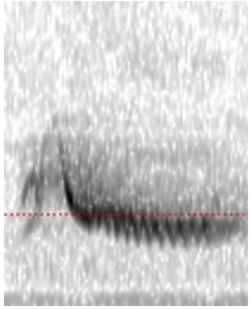

cn

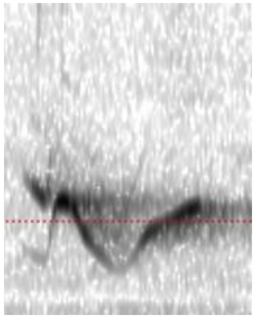

co

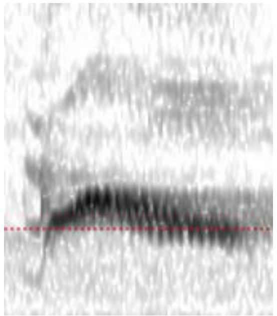

cp

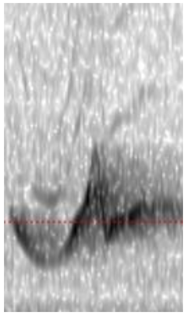

cr

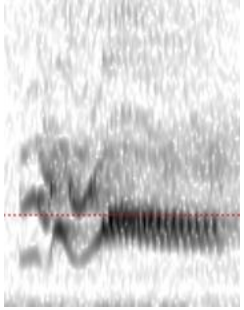
da

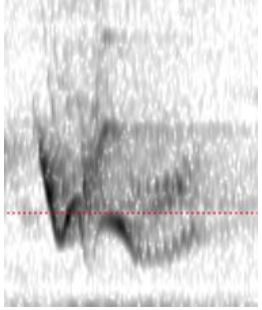
db

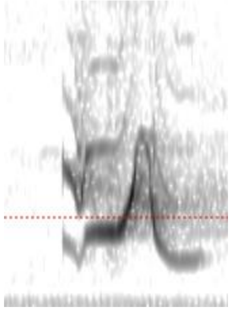
dd

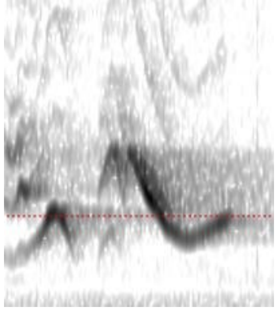
df

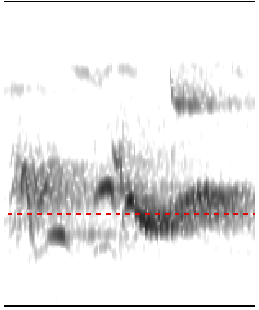
dg

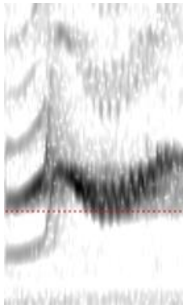
dh

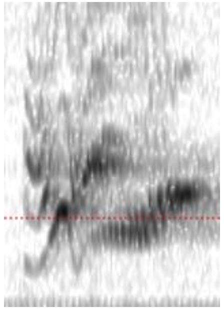
di

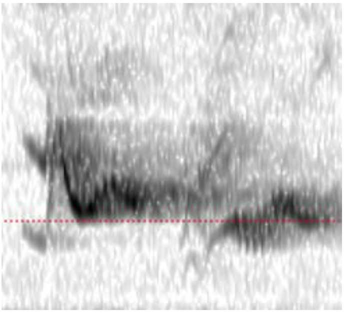
dl

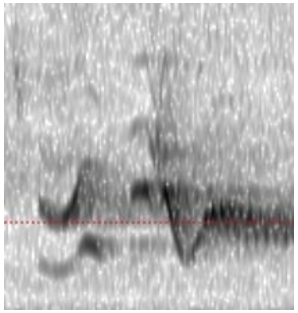
dm

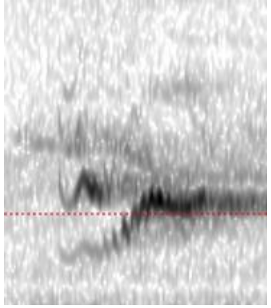
dn

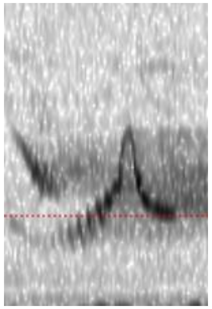
do

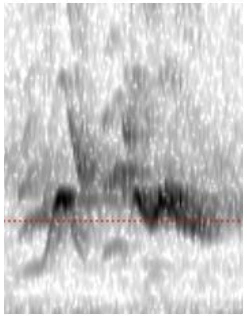
dp

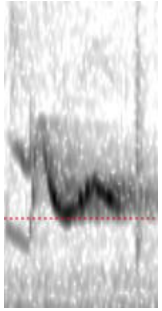
dr

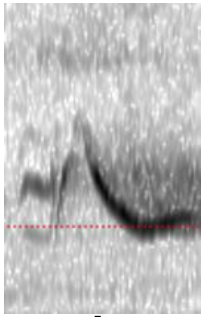
ds

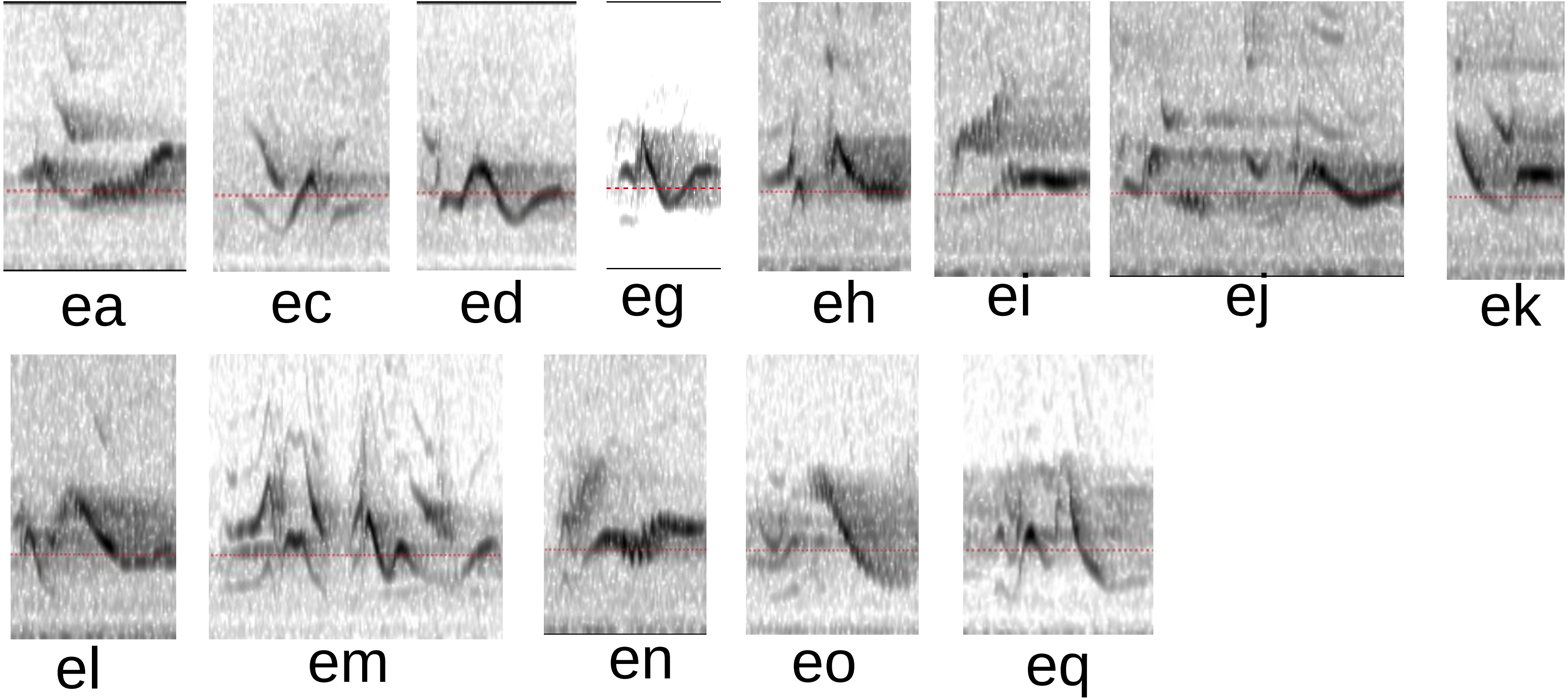
ea
ec
ed
eg
eh
ei
ej
ek
el
em
en
eo
eq

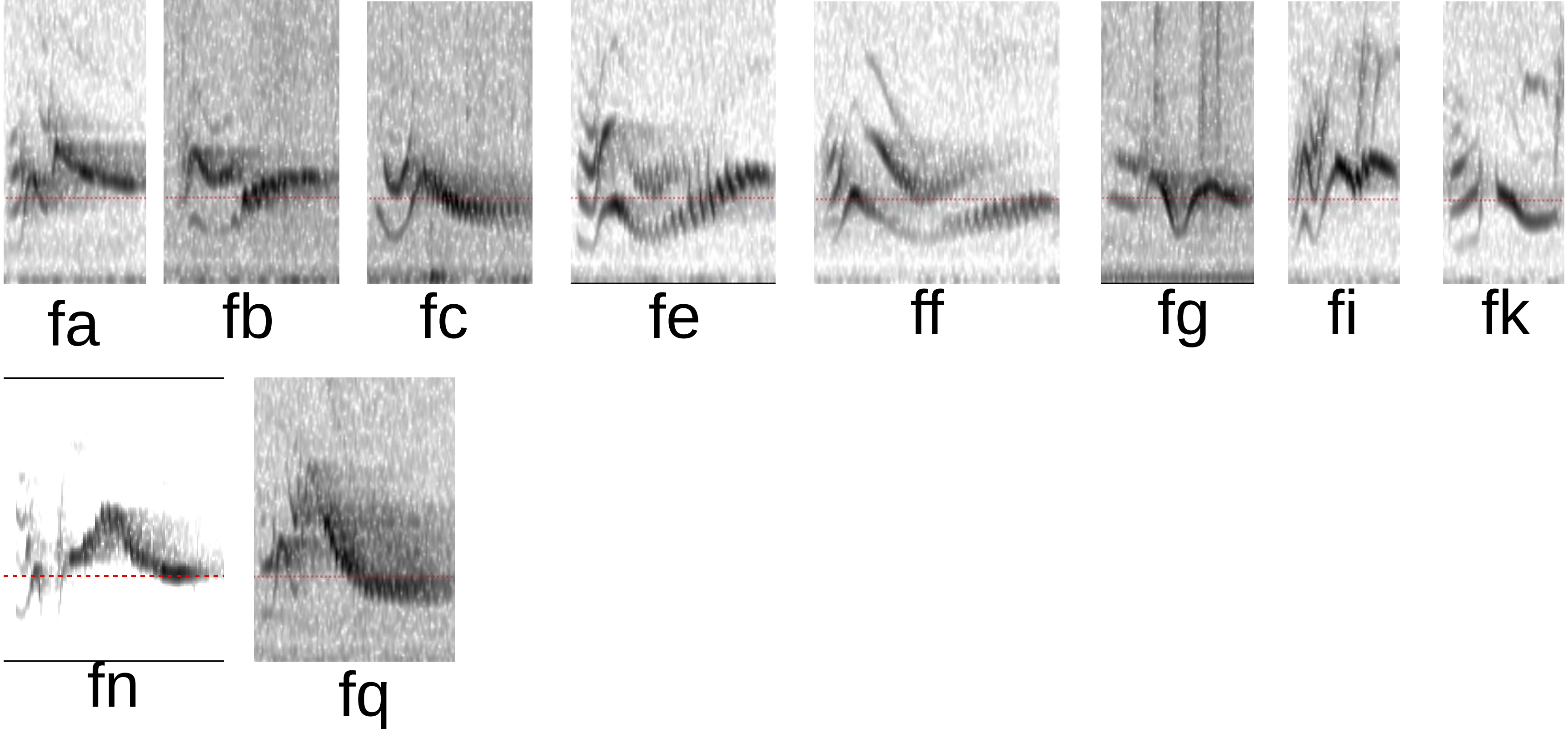
fa
fb
fc
fe
ff
fg
fi
fk
fn
fq

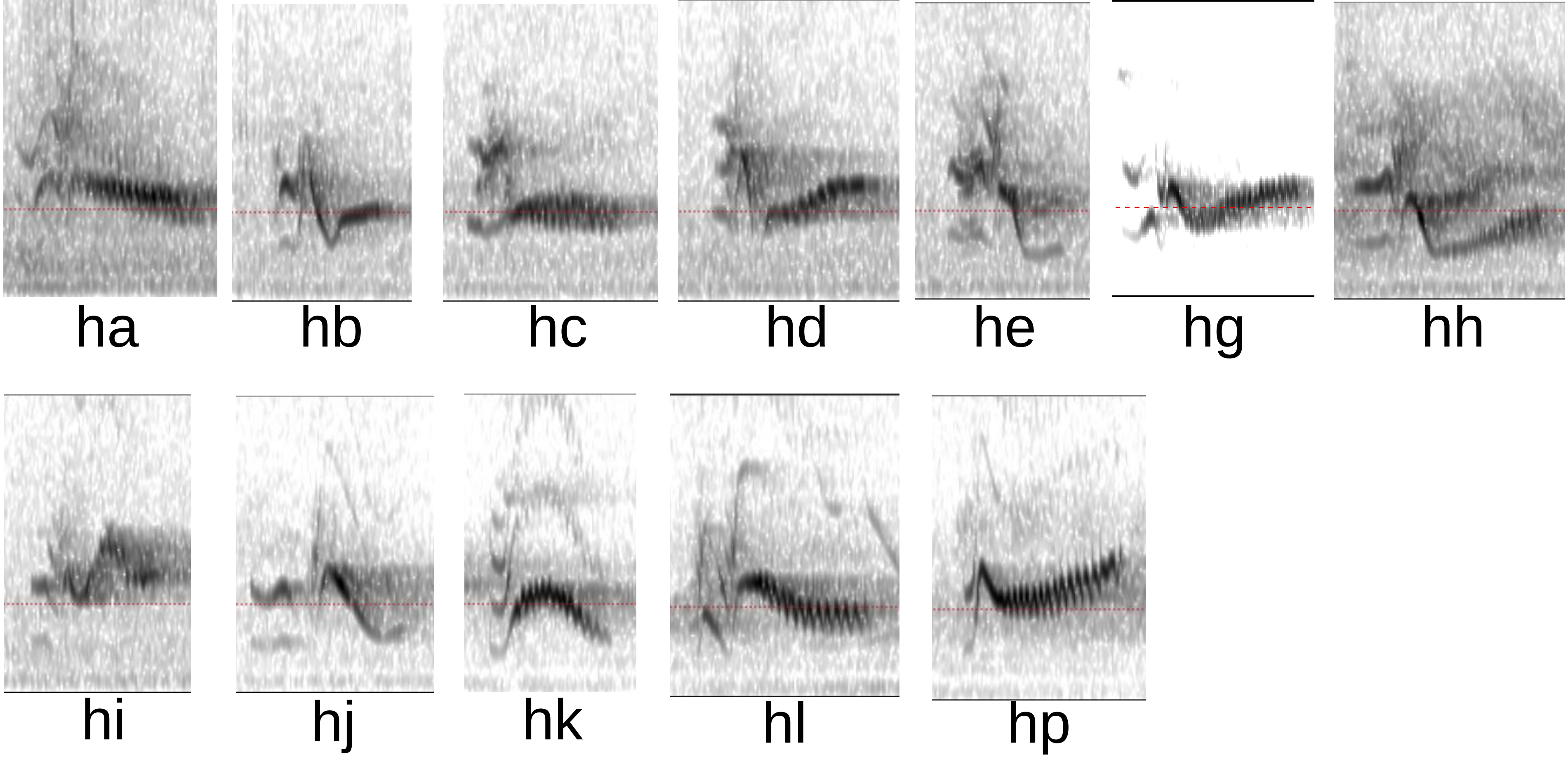
ha
hb
hc
hd
he
hg
hh
hi
hj
hk
hl
hp

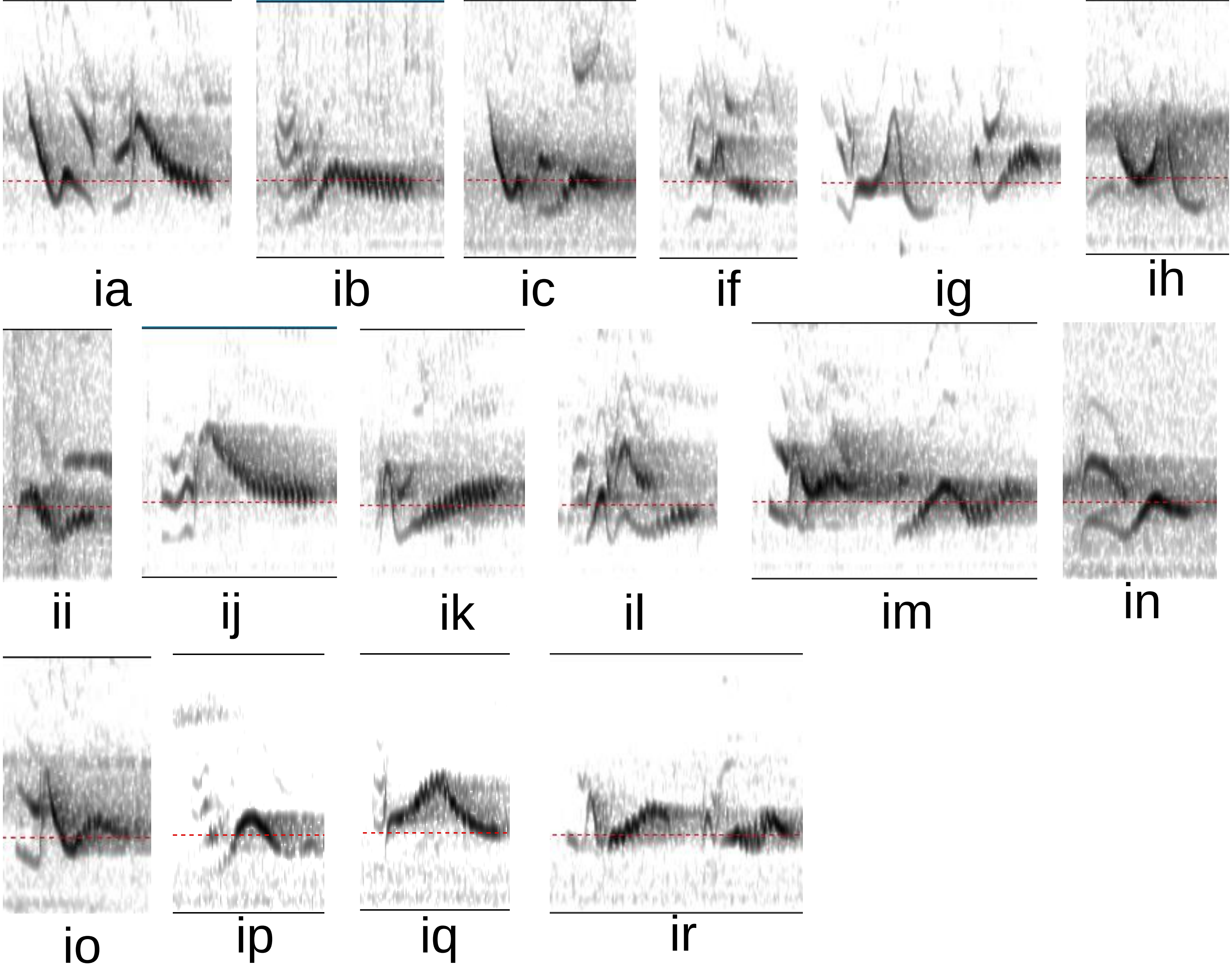
ia
ib
ic
if
ig
ih
ii
ij
ik
il
im
in
io
ip
iq
ir

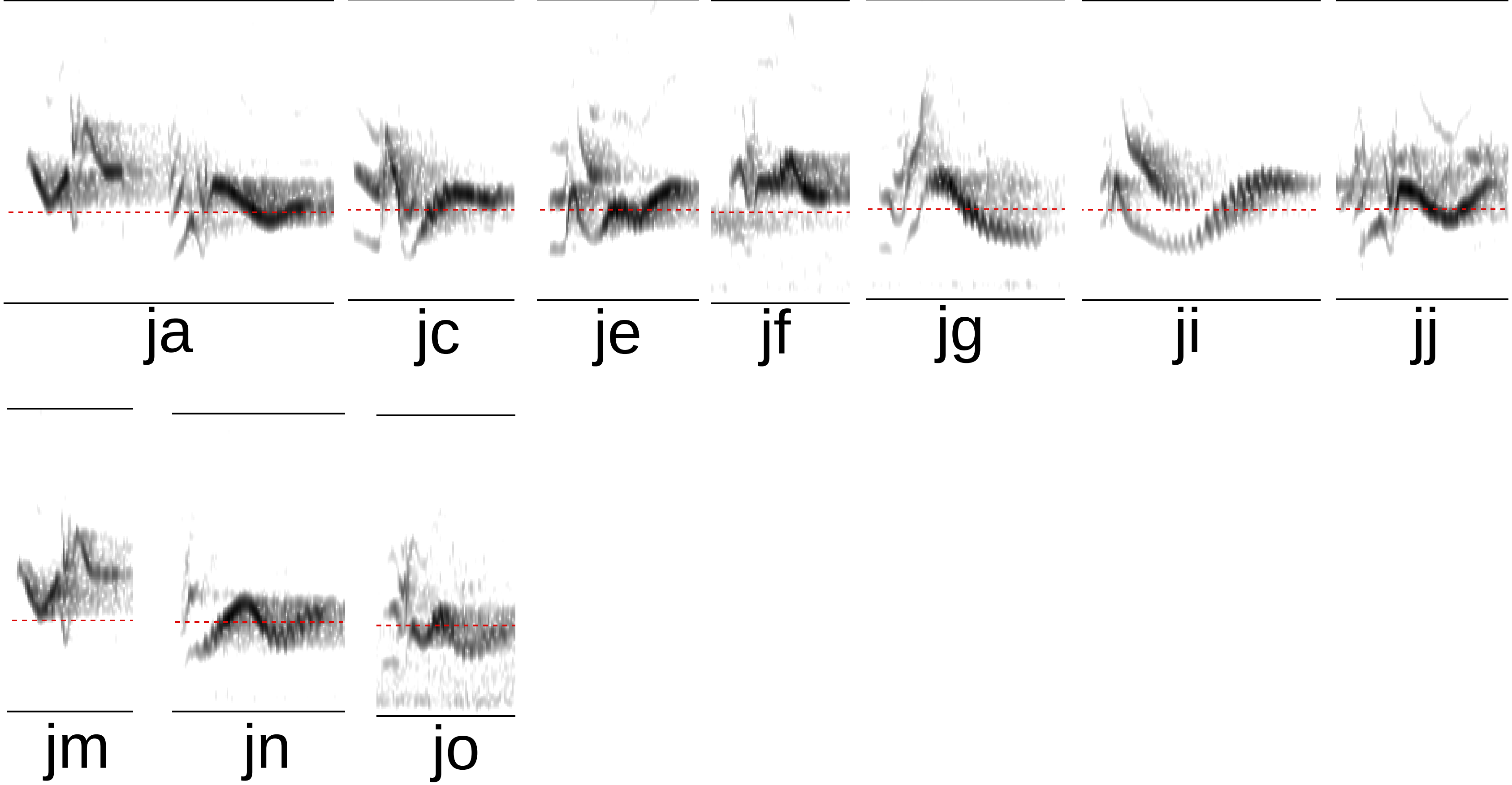
ja
jc
je
jf
jg
ji
jj
jm
jn
jo
